# Supplementary material for: Combined Salivary Proteome Profiling and Machine Learning Analysis Provides Insight into Molecular Signature for Autoimmune Liver Diseases Classification
Source: Int J Mol Sci. 2023 Jul 30;24(15):12207. doi: 10.3390/ijms241512207 (PMC10418803; doi:10.3390/ijms241512207)
Supplement: Supplementary file 1 [file ijms-24-12207-s001.zip › ijms-2481008-supplementary.pdf]

Table S1: Demographic data of 17 AIHp, 17 PBCp and 17 HCs involved in the study.

AIHp	Sex	Age
A02	F	70
A03	F	64
A06	M	45
A12	F	74
A13	F	45
A15	F	83
A16	F	55
A17	F	60
A18	F	45
A20	F	68
A24	F	75
A28	F	57
A31	M	52
A33	F	53
A38	F	40
A42	F	75
A43	F	68

PBCp	Sex	Age
P01	F	60
P02	F	52
P03	F	58
P06	F	65
P07	F	82
P08	F	66
P09	F	64
P10	F	66
P11	F	83
P13	F	56
P14	F	52
P20	F	70
P22	F	55
P24	F	52
P26	F	69
P30	F	61
P41	F	61

HCs	Sex	Age
C05	F	56
C07	F	48
C09	F	41
C11	F	44
C18	F	43
C19	F	63
C22	F	83
C26	F	73
C28	F	78
C33	F	46
C36	F	76
C39	F	51
C40	F	72
C42	F	52
C44	F	54
C60	F	63
C65	F	59

Table S2: Topological features of the network obtained from the 23 proteins varied among HCs, PBCp and AIHp. BC; Betweenness centrality; CC; Closeness Centrality.

Uniprot ID	Gene name	Degree	BC	CC
P00558	PGK1	5	0.712121	0.521739
Q6NUR9	YWHAZ	3	0.590909	0.5
P23528	CFL1	3	0.409090	0.413793
P06396	GSN	3	0.166666	0.324324
P52790	HK3	2	0.166666	0.375
P22626	HNRNPA2B1	3	0.075757	0.387096
P23396	RPS3	3	0.075757	0.387096
P61247	RPS3A	2	0.0	0.292682
P46940	IQGAP1	2	0.0	0.315789
P15924	DSP	2	0.0	1.0
Q08554	DSC1	2	0.0	1.0
P14923	JUP	2	0.0	1.0

P27824	CANX	1	0.0	0.342857
Q86YZ3	HRNR	1	0.0	1.0
P08571	CD14	1	0.0	0.279069
P22528	SPRR1B	1	0.0	1.0
Q8N4F0	BPIFB2	1	0.0	1.0
P10909	CLU	1	0.0	0.25
Q96DR5	BPIFA2	1	0.0	1.0
P68366	TUBA4A	1	0.0	0.352941
P05089	ARG1	0	0.0	0.0
P09758	TACSTD2	0	0.0	0.0
P35237	SERPINB6	0	0.0	0.0

Figure S1. SDS-PAGE of the acidic-insoluble fraction of saliva from HCs, PBCp and AIHp. Each lane was loaded with 10 µg of solubilized proteins. Boxes indicate the three parts of the each lane at different molecular weight (portion A: 250-75 kDa, portion B: 75-25 kDa, portion C: 25 kDa-to the end of gel) that were cut into small pieces with scalpel and submitted to an in-gel digestion.

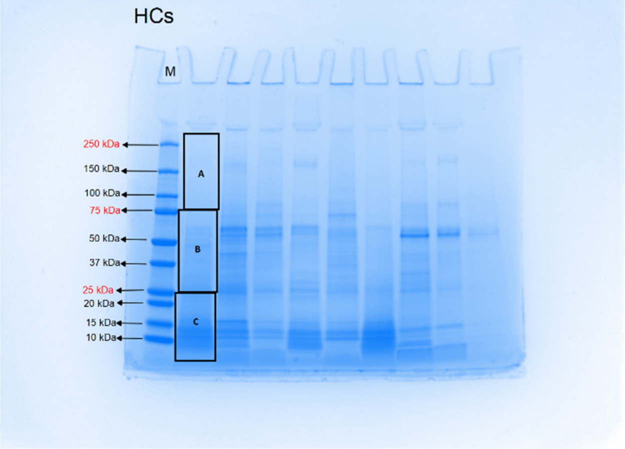

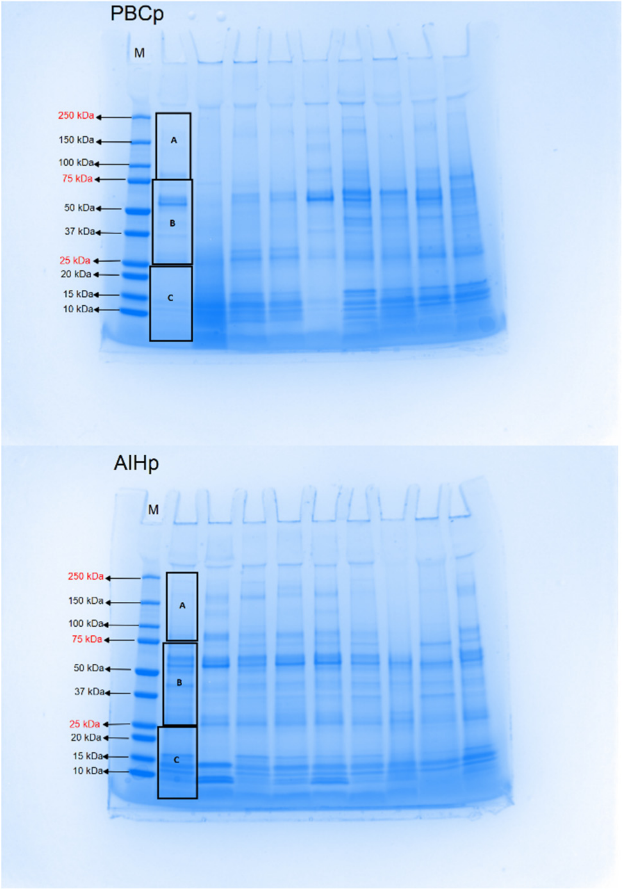
PBCp
M
250 kDa
150 kDa
100 kDa
75 kDa
50 kDa
37 kDa
25 kDa
20 kDa
15 kDa
10 kDa
A
B
C
AlHp
M
250 kDa
150 kDa
100 kDa
75 kDa
50 kDa
37 kDa
25 kDa
20 kDa
15 kDa
10 kDa
A
B
C
